# Supplementary material for: Lactobacillus fermentum HY7302 Improves Dry Eye Symptoms in a Mouse Model of Benzalkonium Chloride-Induced Eye Dysfunction and Human Conjunctiva Epithelial Cells
Source: Int J Mol Sci. 2023 Jun 20;24(12):10378. doi: 10.3390/ijms241210378 (PMC10299454; doi:10.3390/ijms241210378)
Supplement: Supplementary file 1 [file ijms-24-10378-s001.zip › ijms-2432852-supplementary.pdf]

International Journal of *Molecular Sciences*

MDPI

Article

Lactobacillus fermentum HY7302 Improves Dry Eye Symptoms in a Mouse Model of Benzalkonium Chloride-Induced Eye Dysfunction and Human Conjunctiva Epithelial Cells

Kippeum Lee [1,†], Ji Woong Jeong [1], Jae Jung Shim [1], Hyun Sook Hong [2], Joo Yun Kim [1,*] and Jung Lyoul Lee [1,*]

[1] R & BD Center, hy Co., Ltd., 22, Giheungdanji-ro 24 Beon-gil, Giheung-gu, Yongin-si 17086, Republic of Korea; joy4917@hanmail.net (K.L.); woongshow@hy.co.kr (J.W.J.); jjshim@hy.co.kr (J.J.S.)
[2] Kyung Hee Institute of Regenerative Medicine (KIRM), Medical Science Research Institute, Kyung Hee University Medical Center, Seoul 02447, Republic of Korea; hshong@khu.ac.kr
* Correspondence: monera@hy.co.kr (J.Y.K.); jlleesk@re.yakult.co.kr (J.L.L.)
† First author.

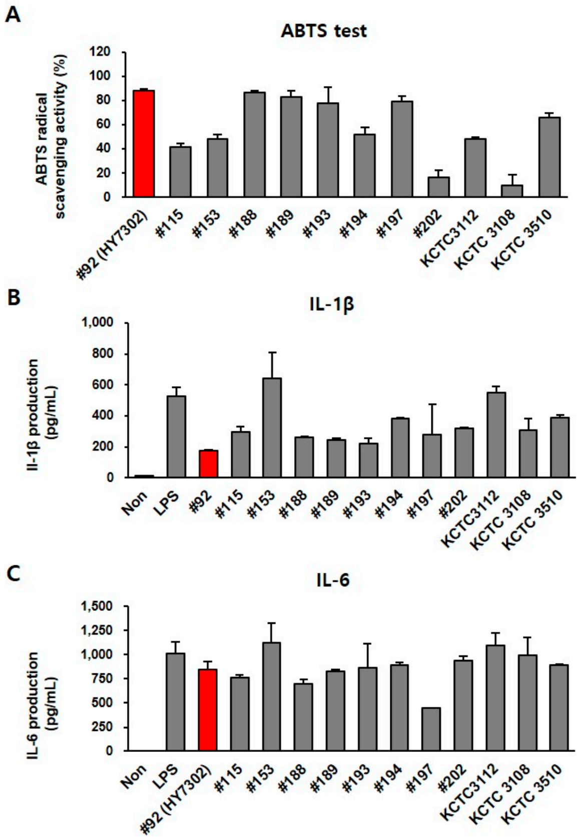

Supplementary Figure S1. ABTS radical scavenging activity and pro-inflammatory cytokine inhibitory effect of candidate lactic acid bacteria in RAW264.7 macrophages. (**A**). Cells were pretreated with candidate lactic acid bacteria and 100 ng/mL lipopolysaccharides (LPS) for 24 h. (**B**) IL-1β and (**C**) IL-6 production were measured using enzyme-linked immunosorbent assay (ELISA) kit.
